# Prevalence, awareness, treatment and control of high blood pressure in the elderly according to the ethnic group. Colombian survey

**DOI:** 10.25100/cm.v50i2.4124

**Published:** 2019-06-30

**Authors:** Lena Barrera, Fernando Gómez, Delia Ortega-Lenis, Jairo Corchuelo Ojeda, Fabián Méndez

**Affiliations:** 1 Universidad del Valle, Facultad de Salud, Escuela de Medicina.Cali, Colombia.; 2 Universidad del Valle, Facultad de Salud Escuela de Salud Pública, Cali, Colombia; 3 Universidad de Caldas, Facultad de Ciencias para la Salud, Cali, Colombia; 4 Universidad del Valle, Escuela de Salud Pública, Grupo epidemiología y salud poblacional (GESP), Cali, Colombia; 5 Pontificia Universidad Javeriana, Departamento de Salud Pública y Epidemiología, Cali, Colombia; 6 Universidad del Valle, Facultad de Salud, Escuela de Odontología, Cali, Colombia

**Keywords:** Hypertension, blood pressure, older adults, elderly, ethnicity, skin pigmentation, ethnic groups, cardiovascular diseases, health surveys, aging, aged, surveys and questionnaires, Colombia., Hipertensión, presión sanguínea, adulto mayor, viejo, etnia, pigmentación de la piel, grupos etnicos, enfermedades cardiovasculares, encuesta en salud, envejecimiento, vejez, encuesas y cuestionarios, Colombia

## Abstract

**Introduction:**

High blood pressure (HBP) is the main cardiovascular risk factor, it is more prevalent in the older adult population, and the prevalence can vary between ethnic groups.

**Objective:**

To estimate the prevalence of HBP, knowledge, treatment and control in population aged ≥60 years, resident in Colombia, according to their ethnic condition.

**Methods:**

Population sample selected by multistage sampling. Ethnicity was defined based on skin color. HBP was defined as systolic blood pressure ≥140, and/or diastolic blood pressure ≥90 mm Hg, and/or the participants’ self-report. Controlled HBP at a blood pressure value <140/90, knowledge and treatment were identified by self-report.

**Results:**

23,694 adults aged ≥ 60 years were included, of which 54.5%, 34.5% and 10.9% were respectively identified as having light, medium or dark skin color; 54.5% were women, and 78.1% resided in urban areas. The standardized prevalence of HBP, by age, was 57.7% (95% CI: 55.2 - 60.2); 51.4% (95% CI: 47.3-55. 5), in men; and 62.9% (60.9-64.9), in women. The standardized prevalence for light, medium and dark skin in men was 53.2% (95% CI: 48.7-57.7), 49.6% (44.5-54.7), and 49.4% (95% CI: 41.0-57.8) respectively; and in women was 62.5% (95% CI: 60.5-64 , 5), 61.7% (95% CI: 57.8-65.6), and 69.9% (95% CI: 63.6-76.2) respectively. 98% of the population received treatment, and 93.9% were aware of HBP diagnosis. 42.5% of men and 55.5% of women with HBP were under control. Only 21.8% performed regular physical activity.

**Conclusion:**

Half of the adult population aged over 60 years suffers from HBP; the prevalence is higher in women particularly in dark-skinned women. It is necessary to develop policies to increase physical activity in the elderly.

## Resumen

**Introducción:**

La presión arterial elevada (PAE) es el principal de factor de riesgo cardiovascular, es más prevalente en población adulta mayor y la prevalencia puede variar entre etnias.

**Objetivo:**

Estimar la prevalencia de PAE, conocimiento, tratamiento y control en población ≥60 años residente en Colombia de acuerdo con su condición étnica.

**Métodos:**

Muestra poblacional seleccionada mediante un muestreo multietápico. La etnia se definió con base en el color de piel. La PAE se definió como presión arterial sistólica ≥140 y/o presión arterial diastólica ≥90 mm Hg y/o el autoreporte del participante. La PAE controlada a un valor de presión arterial <140/90, el conocimiento y el tratamiento se identificaron por autoreporte.

**Resultados:**

Fueron incluidos 23,694 adultos con edad ≥ 60 años, de los cuales, 54.5%, 34.5% y 10.9% se identificaron con color de piel claro, medio u oscuro respectivamente, El 54.5%, eran mujeres y el 78.1% residía en zona urbana, La prevalencia estandarizada por edad de PAE fue 57,7% (IC 95%: 55.2-60.2), 51.4% (IC 95%: 4.3-55.5) en hombres y 62.9% (60.9-64.9) en mujeres, La prevalencia estandarizada en hombres claros, medios y oscuros fue 53.2%(IC 95%: 48.7-57.7), 49.6% (44.5-54.7) y 49.4% (IC 95%: 41.0-57.8) respectivamente y en mujeres 62.5% (IC 95%: 60.5-64.5), 61.7% (IC 95%: 57.8-65.6) y 69.9% (IC 95%: 63.6-76.2) respectivamente, El 98% de la población recibía tratamiento y el 93.9% conocía el diagnóstico de PAE, El 42.5% de los hombres y el 55.5% de las mujeres se encontraban con PAE controlada, El 21.8% realizaba actividad física regularmente.

**Conclusión:**

La mitad de la población adulta mayor a 60 años sufre PAE, mayor prevalencia en mujeres y particularmente mujeres oscuras. Se requiere desarrollar políticas para incrementar la actividad física en el adulto mayor.

**Remark**

**Table:** 

1)Why was this study done?
To estimate the prevalence of raised blood pressure (RBP) in Colombian people aged 60 years and older according with skin color.
**2) What did the researchers do and find?**
We carried out a national survey of older people in 2014. 57,7% of older people had RBP with a higher prevalence in women with black skin colour, 60,9%. 93.9% of those with RBP were aware of their diagnosis and among those with treatment 50,1% had controlled RBP. There were no differences in control and awareness between people regarding skin colour. Low rates of physical activity were found in all people
**3) What do these findings mean?**
RBP is highly prevalent in Colombian older people and only half had controlled RBP. More efforts are needed to prevent RBP and to improve the management of RBP in the elderly. Particulary, initiatives to improve physical activity are required

## Introduction

High blood pressure (HBP) is the main cardiovascular risk factor worldwide ^1^. For 2015, global estimates of the prevalence of HBP (i.e., blood pressure (BP) ≥140/90 mm Hg) in the population aged over 18 years were 24.1% for men and 20.1% for women ^2^. BP increases with age, particularly in Western societies, to the point that it has been reported that about 45% of the population aged over 60 years suffers from HBP ^3^. The prevalence differs between countries; in some like Mexico, a prevalence of 58.2% has been reported; while in India, the estimated prevalence is 32.3% in population aged over 50 years.

Differences in the prevalence of HBP have been reported among ethnic groups ^4^. The United States Health Survey --National Health and Nutrition Examination Survey (NHANES)-- has consistently reported a higher prevalence of HBP in the non-Hispanic black population, compared to the non-Hispanic white population 40.3% and 27.8% respectively in 2016 ) ^4^ ^,^ ^5^. However, this higher prevalence was not observed in native populations residing in rural communities in Africa. For example, a lower prevalence of HBP was estimated in native populations residing in Ghana (4.6%) and Jamaica (6.8%), compared to the Afro-descendant population living in the United States (29.0%) ^6^. Similarly, in Brazil, a slightly higher prevalence of HBP was found in the black population (25.8%), compared to the white population (24.1%) ^7^. On the contrary, in Cuba, a similar prevalence of HBP has been observed in black and white populations, 31% ^8^. All these observations suggest health inequities and provide evidence on the influence of social determinants on the development of HBP, particularly in the African diaspora, in contrast to the genetic explanations associated with the presence of HBP in that population ^6^.

In Colombia, considered as a middle-income country, the last estimated national prevalence of HBP was 23.7% for the population aged between 18 and 69 years in 2007 ^9^. The prevalence increased with age, being higher in the population aged between 60 and 69, 58.9% ^9^. Similarly, in Bogotá (SABE-Bogotá), a prevalence of 56.9% was reported in people aged ≥60 years ^10^. On the other hand, the international study of mobility in aging (IMIAS) found a prevalence of HBP near to 70% in a population aged between 64 and 75 years residing in an intermediate city located in the central area of ​​the country ^11^. None of these surveys considered ethnic characteristics in relation to the prevalence of HBP.

The National Administrative Department of Statistics of Colombia (DANE, for its initials in Spanish) has established four ethnic groups, based on self-recognition: Indigenous; Afro-Colombian; *Raizal*, ancestral people from the San Andres, Providencia and Santa Catalina archipelago; and *Roma* people (singular Rom, also called Romany, or *Gypsies*, being the last considered an offensive term - Encyclopaedia Britannica). In particular, the Afro-Colombian population, defined as persons with recognized African ancestry, includes the *Palenqueros* of *San Basilio*, blacks and/or mulattos, representing 10.6% of the Colombian population, according to the census carried out in 2007 ^12^. People who do not identify themselves within these groups are considered without ethnic self-recognition ^13^. The most frequently used classification to identify ethnicity/race is based on people's self-recognition; however, it has been determined that the external classification by an interviewer of skin color has a close correlation with ethnic and cultural characteristics, and accounts for other aspects not considered by self-recognition. This observation has led to the development of a scale or color palette instrument to determine the ethnicity/race of a person ^14^. 

The Afro-descendant ethnic population in Colombia resides primarily in the Pacific and Caribbean region; Valle del Cauca province has the highest proportion of residents identified as Afro-Colombians, 25.3% ^12^, while the indigenous population can be mainly found in the provinces of Orinoquia, Amazonia and Chocó. The morbidity and mortality studies related to HBP available so far do not report results according to the ethnic characteristics of the population ^15^. In consideration of the above, the data of the Colombian Health, Welfare and Aging (SABE) survey, carried out in 2015, was analyzed in order to estimate the prevalence of high blood pressure, knowledge, treatment and control of this condition in the population aged 60 years or older in the ethnic groups identified in the survey.

## Materials and Methods

The SABE survey aimed to establish the state of health, well-being and social conditions of the Colombian population aged 60 years or older in the community. The survey methods were previously described ^16^. In the survey, the variables were grouped according to the categories of determinant factors of active aging, as follows: economic, social environment, physical environment, personal factors, behavior, and social and health services systems. In addition, since active aging includes the role of culture and gender as transversal factors that influence all other determinants, the SABE study included analysis variables related to demographic indicators, socioeconomic status, geographic regions and race/ethnicity. To identify the socioeconomic stratum, the classification of strata of the public utilities was used; the social security regime was determined taking into account the type of affiliation reported, according to the categories contributory, subsidized, exception or special regime. When the person said (that) he/she was not affiliated, it was inquired about the reasons for non-affiliation.

In addition, there were evaluated habits such as smoking cigarettes (if they did t or not, if you were currently doing it, time in years of smoking, age when they started smoking, amount of cigarettes or tobacco they smoke or smoked per day) and alcohol consumption (current and past behavior). Current physical activity was assessed by adapting the scale of daily activities of Reuben's daily life ^17^. Participants were classified into four categories, according to their responses: vigorous frequent athletes, frequent walkers, frequent short walkers, and people who did not exercise frequently (sedentary group). Nutritional status was assessed using the longest original version of the Mini-Nutritional Assessment (MNA) ^18^. Anthropometry measures include height and body weight; there were used a portable stadiometer (SECA 213), and an electronic scale (graduated Kendall platform scale). 

In a novel way, in addition to the questions of ethnic and racial self-recognition traditionally included in this type of study, the survey used an external classification conducted by an interviewer who used a color palette (See supplementary material). The procedure for using the palette has been widely validated in Latin America in the PERLA and LAPOP surveys ^14^ ^,^ ^19^. Specifically, the interviewer classified the face of the person interviewed in one of the eleven colors included in the palette, 1 being the lightest color and 11 the darkest color. For the analysis of this variable, the population was categorized, according to the palette scale, into three groups: a) light skin color (1 to 3); b) intermediate (4 to 5); and c) dark (6 to 11). 

The sample was taken based on the reference population of the master sample of national surveys, using a multi-stage sampling technique, stratified by clusters. In the analysis, different sample weights were used, according to the population estimated by DANE for 2015, in order to consider the probabilities of selection of the sample design. A person with HBP was defined as one who met at least one of the following criteria: a) to refer a diagnosis of HBP, which had been made by a health worker; or b) to refer the use of medications to reduce blood pressure; or c) to have a measurement of systolic blood pressure (SBP) greater than or equal to 140, and/or a diastolic blood pressure (DBP) greater than or equal to 90 mm Hg. The blood pressure was measured in a subsample of 5,760 people aged over 60 years, and the value used for the classification was the average of the last two measurements made, according to the established protocol ^16^ ^,^ ^19^ ^,^ ^20^.

The overall prevalence of HBP was estimated using the direct standardization method, using as a reference the Colombian population projected by DANE for 2015 ^21^ ^,^ ^22^. The prevalence of knowledge about suffering HBP was estimated as the fraction among those who reported the diagnosis by a health worker and/or were taking medicines to reduce BP, and the total population classified with HBP ^23^. The prevalence in people who referred to be receiving treatment was determined among the population classified with HBP; while the prevalence of control was determined as the ratio between hypertensive people treated with BP <140/90 mm Hg and the total number of people with HBP who reported taking medications to reduce BP ^20^. Estimates were made for each of the groups that were defined based on skin color. Only variable categories with a relative standard error (EE) <30% were included in the analyses ^20^. The variance was estimated using the Taylor series linearization method ^23^. The analysis was performed with the *STATA v. 14* program. 

## Results

A total of 23,694 adults aged ≥60 years were included, based on the estimated sample. Of that total, 54.5%, 34.5% and 10.9% were classified with light-, medium- and dark-skinned, respectively. Of the entire population included, a higher proportion was found in the age group between 60 and 69 years, 56.9% (95% CI: 55.5 - 58.3); 54.5% were female (95% CI 53.3 -55.8); 78.1% lived in an urban area (63.2 - 88.1); 27.0% lived in the downtown area (95% CI 9.3- 57.2); 20.8% belonged to a low socio-economic strata, specifically in stratum 1 (CI 95 % 19.5 - 39.3); and 38.6% to stratum 2 (95% CI 36.5-42.9). Only 2.2% (95% CI 1.6-3.0) of the population was not affiliated with any of the plans of the Colombian social security regime. Compared to the population classified with light-, medium- or dark-skinned, a higher percentage of population classified with dark skin color was found in the low socio-economic stratum, lived in rural areas, and was affiliated with the subsidized regime. 8% of the population with dark skin resides in Cali, being the main city with the highest concentration of population with that skin color (Table 1). 

**Table 1:** Characteristics of the total population included distributed by skin color

Characteristics		Light		Medium		Dark		Total	
	n*	%	IC**	%	IC**	%	IC**	%	IC**
Sex									
Men	10,112	41.0	39.1-42.8	49.8	46.1-53.4	54.2	50.2-58.2	45.5	44.2-46.7
Women	13,582	59.0	57.2-60.9	50.2	46.6-53.9	45.8	41.8-49.8	54.5	53.3-55.8
**Age (years)**									
60-69	12,101	56.0	54.1-57.9	58.5	56.9-60.1	56.3	52.4-60.1	56.9	55.5-58.3
70-79	7,720	30.5	28.7-32.4	29.4	27.8-31.1	31.0	26.9-35.4	30.2	29.1-31.4
≥ 80	3,873	13.5	12.3-14.8	12.1	10.9-13.4	12.8	11.2-14.5	12.9	12.2-13.7
**Socio-economic stratum**									
1	10,313	20.8	13.7-30.3	33.0	23.7-43.8	51.9	42.5-61.2	28.4	19.5-39.3
2	9,033	38.6	36.2-41.1	43.0	37.4-48.7	34.3	28.3-40.9	39.7	36.5-42.9
3-4	4,181	37.7	28.8-47.4	22.8	17.4-29.3	13.6	8.7-20.5	29.9	22.0-39.3
5- 6	167	2.9	1.6-5.1	1.2	0.6-2.4	0.2	0.1-0.6	2.0	1.3-3.2
**Area**									
Urban	17189	84.5	70.0-92.7	72.3	55.7-84.4	65.0	47.2-79.5	78.1	63.2-88.1
Rural	6505	15.5	7.3-30.0	27.7	15.6-44.3	35.0	20.5-52.8	21.9	11.9-36.8
**Region**									
Atlántico	6202	10.6	3.5-27.9	24.5	11.2-45.5	44.5	23.7-67.4	19.1	8.0-39.1
East	3583	20.7	7.5-45.6	18.2	7.0-39.6	3.3	0.9-11.4	17.9	6.9-39.1
Orinoquia and Amazonia	1394	1.5	0.4-5.5	1.4	0.4-4.7	0.5	0.2-1.1	1.4	0.4-4.5
Bogotá	2003	23.4	3.6-71.2	11.2	1.6-49.9	3.7	0.5-23.4	17.0	2.5-62.0
Central	6351	29.5	9.8-61.8	27.0	9.5-56.6	15.0	4.2-41.4	27.0	9.3-57.2
Pacific	4161	14.4	3.2-46.3	17.6	5.9-42.4	33.1	14.5-59.1	17.5	5.2-45.4
**Capital cities**									
Barranquilla	544	3.1	0.4-20.5	4.1	0.6-24.8	5.5	0.8-30.3	3.7	0.5-23.2
Cali	923	7.4	1.0-38.3	5.7	0.8-30.8	8.1	1.3-37.0	6.9	1.0-35.5
Medellín	849	10.3	1.5-46.3	9.1	1.4-41.2	5.0	0.7-27.8	9.3	1.4-42.6
Others	21378	79.2	51.0-93.3	81.1	58.1-93.0	81.4	60.4-92.6	80.1	55.2-92.9
**Social security regime**									
Contributory	8627	56	48.8-62.9	43.4	36.3-50.7	30.8	23.3-39.6	48.9	41.0-56.8
Subsidized	14,160	39.5	32.2-47.3	52.9	45.7-59.9	64.9	56.8-72.3	46.9	38.9-55.1
Special regimes	371	2.6	1.6-4.2	1.2	0.7-1.9	1.4	0.7-2.8	2.0	1.2-3.2
Non-affiliation	512	1.9	1.3-2.7	2.5	1.5-3.9	2.7	1.9-4.1	2.2	1.6-3.0

* N= number

Of the total number of people included, 13,360 people were identified with HBP, 95.0% by interview and 5.0% through the BP measurement. The standardized prevalence by age was 57.7% (95% CI 55.2-60.2); 51.4% (95% CI 47.3-55.5) in men, and 62.9% (95% CI 60.9-64.9) in women. The prevalence increased with age, without finding significant differences by skin color within the age groups studied. However, in the age group ≥80 years, dark-skinned men have a slightly lower prevalence, compared with light-skinned population; on the contrary, in dark-skinned women, the prevalence was higher. Among the population with HBP and record of BP pressure, the average SBP and DBP were 141.0 mm Hg (134.1 -147.9) and 80.0 mm Hg (78.0 - 82.0) respectively. Overall, the prevalence was significantly higher in women, compared to men: 62.9% (95% CI 60.9 - 64.9) vs. 51.4% (95% CI 47.3, -55.5) (Table 2).

**Table 2:** Standardized prevalence * of high blood pressure by age for each sex and skin color.

	60-69 years		70-79 years		≥80 years		Total	
	%	CI‡	%	CI	%	CI	%	CI
**Men**								
Light	45.0	39.3-50.7	61.8	56.1-67.5	70.6	64.1-77.1	53.2	48.7-57.7
Medium	42.7	35.8-49.6	59.7	52.8-66.6	61.0	54.1-67.9	49.6	44.5-54.7
Dark	41.6	30.8-52.4	60.1	49.3-70.9	59.2	48.4-70.0	49.4	41.0-57.8
Total	43.7	38.4-49.0	60.8	55.9-65.7	65.6	58.9-72.3	51.4	47.3-55.5
**Women**								
Light	58.3	55.8-60.8	65.5	63.0-68.0	71.9	66.8-77.0	62.5	60.5-64.5
Medium	55.2	48.9-61.5	67.4	61.1-73.7	76.9	71.6-82.2	61.7	57.8-65.6
Dark	65.0	57.2-72.8	73.6	65.8-81.4	80.4	74.3-86.5	69.9	63.6-76.2
Total	57.9	55.2-60.6	66.9	64.2-69.6	74.2	70.5-77.9	62.9	60.9-64.9
General Total	51.2	47.7-54.7	64.2	61.5-66.9	70.6	66.3-74.9	57.7	55.2-60.2
Diastolic blood pressure levels**								
	**Mean value**	**CI**	**Mean value**	**CI**	**Mean value**	**CI**	**Mean value**	**CI**
**Men**								
Light	82.1	80.9-83.3	81.9	79.5-84.3	74.8	71.5-78.1	81.1	79.7-82.5
Medium	84.7	81.0-88.4	80.2	78.0-82.4	78.8	74.3-83.3	82.8	79.9-85.7
Dark	83.6	78.7-88.5	79.3	75.2-83.4	79.1	76.0-82.2	81.7	78.6-84.8
Mean value	83.3	81.5-85.1	81.1	79.1-83.1	76.8	73.9-79.7	81.8	80.2-83.4
**Women**								
Light	79.8	75.9-83.7	78.7	76.5-80.9	75.0	72.8-77.2	78.9	76.2-81.6
Medium	78.6	76.4-80.8	78.0	75.8-80.2	75.7	74.1-77.3	78.0	76.4-79.6
Dark	80.8	77.5-84.1	79.6	75.1-84.1	77.1	71.4-82.8	79.7	76.8-82.6
Mean value	79.5	76.2-82.8	78.5	76.7-80.3	75.4	74.0-76.8	78.7	76.3-81.1
General mean value	81.2	78.7-83.7	79.7	77.9-81.5	76.0	74.0-78.0	80.0	78.0-82.0
Systolic blood pressure levels **								
	**Mean value**	**CI**	**Mean value**	**CI**	**Mean value**	**CI**	**Mean value**	**CI**
**Men**								
Light	136.9	128.9-144.9	149.6	141.4-157.8	145.9	132.2-159.6	142.3	133.5-151.1
Medium	144.2	136.6-151.8	141.4	134.1-148.7	140.9	129.3-152.5	143.1	136.8-149.4
Dark	141.9	131.7-152.1	145.8	135.2-156.4	152.9	141.3-164.5	144.5	135.3-153.7
Mean value	140.3	133.8-146.8	146.8	139.5-154.1	144.5	136.1-152.9	142.8	136.1-149.5
**Woman**								
Light	135.7	127.7-143.7	143.9	135.7-152.1	142.5	132.3-152.7	139.0	131.4-146.6
Medium	132.7	125.1-140.3	147.5	141.2-153.8	150.4	145.7-155.1	139.5	132.4-146.6
Dark	152.7	135.3-170.1	142.0	131.0-153.0	141.5	132.7-150.3	147.3	135.7-158.9
Mean value	135.7	127.9-143.5	144.8	137.7-151.9	144.8	136.4-153.2	139.6	132.3-146.9
General mean value	137.7	130.6-144.8	145.7	138.8-152.6	144.7	137.4-152	141.0	134.1-147.9

* Standardized prevalence using the direct method and the Colombian population, 2015

** Pressure levels in mm Hg estimated for the subsample

The percentage of hypertensive people who were aware of their condition was 93.9% (95% CI: 90.8, - 95.9), with no difference between the sexes. In the population with light and medium skin color, the groups aged less than 80 years had a lower prevalence of awareness, compared to the population aged over 80 years. On the contrary, a significant lower proportion of men reported using antihypertensive drugs, compared to women; overall, 88.8% (95% CI: 86.8-90.5) of the population with HBP received treatment. Regardless of skin color, the lowest percentage of treatment was found in men aged between 60 and 69 years. Overall, among men, the group of medium-skinned men presented a lower prevalence of treatment, compared with light-skinned men. On the other hand, among women, a smaller proportion of dark-skinned women were treated, compared with light-skinned women (Table 3).

**Table 3:** Awareness, treatment and control of people aged 60 years or older with high blood pressure

Percentage of people who were aware about being hypertensive								
	60-69		70-79		≥ 80		Total	
	%	CI*	%	CI	%	CI	%	CI
**Age group**								
**Men**								
Light	90.4	82.0-95.1	91.4	85.3-95.1	91.0	85.6-94.5	90.8	85.5-94.3
Medium	87.1	78.3-92.6	94.3	90.4-96.6	96.0	91.5-98.2	90.8	85.2-94.4
Dark	92.8	84.9-96.7	94.5	88.0-97.5	93.4	81.7-97.8	93.5	88.9-96.3
Total	89.4	83.0-93.6	92.8	89.4-95.2	93.0	89.4-95.4	91.1	87.0-94.1
**Women**								
Light	94.4	87.6-97.6	95.5	93.0-97.2	97.5	95.2-98.7	95.3	91.9-97.3
Medium	97.9	96.5-98.8	94.0	86.4-97.5	94.3	86.9-97.6	96.1	93.1-97.8
Dark	95.2	86.9-98.4	98.2	93.6-99.5	100.0	0.0-0.0	97.0	92.4-98.8
Total	95.6	90.9-97.9	95.3	92.7-97.0	96.8	94.4-98.2	95.7	93.1-97.4
General Total	93.1	88.3-96.0	94.3	91.8-96.0	95.3	93.2-96.8	93.9	90.8-95.9
**Percentage of hypertensive people who were receiving antihypertensive treatment**								
	**%**	CI*	**%**	**CI**	**%**	**CI**	**%**	**CI**
**Men**								
Light	80.8	76.2-84.7	91.6	87.2-94.6	93.7	88.2-96.7	86.7	84.1-88.9
Medium	79.8	74.4-84.3	81.8	67.5-90.6	80.8	63.4-91.1	80.6	74.4-85.7
Dark	79.6	68.4-87.6	92.4	86.1-96.0	77.1	53.6-90.8	84.2	77.6-89.1
Total	80.3	76.1-83.9	88.2	81.9-92.5	87.3	79.7-92.3	84.2	81.2-86.8
**Women**								
Light	90.3	85.3-93.7	95.5	93.5-96.8	96.6	95.2-97.6	93.0	90.6-94.9
Medium	89.0	86.5-91.1	92.2	84.4-96.3	92.2	87.7-95.2	90.6	88.2-92.5
Dark	82.0	74.0-87.9	93.2	87.8-96.3	96.5	93.1-98.2	88.2	83.4-91.8
Total	89.0	85.9-91.5	94.2	91.0-96.3	95.3	93.6-96.6	91.8	89.7-93.4
General Total	85.7	83.8-87.4	91.7	88.1-94.3	92.3	89.3-94.5	88.8	86.8-90.5
**Percentage of hypertensive people who were receiving treatment and who had blood pressure <140/90 mm Hg**								
	**%**	CI*	**%**	**CI**	**%**	**CI**	**%**	**CI**
**Men**								
Light	46.4	36.9-56.1	32.7	24.9-41.7	41.5	23.2-62.5	40.3	32.0-49.2
Medium	41.8	23.6-62.4	49.6	32.1-67.2	52.1	32.1-71.5	45.2	29.5-62.0
Dark	54.6	23.6-82.3	34.5	10.1-71.1	27.5	8.4-61.0	44.5	18.0-74.5
Total	45.4	33.1-58.2	37.6	27.7-48.6	43.4	30.2-57.5	42.4	31.5-54.1
**Women**								
Light	63.5	52.9-73.0	45.8	31.6-60.8	51.8	37.0-66.4	56.5	46.0-66.4
Medium	63.8	45.9-78.5	48.6	36.5-60.9	31.5	22.4-42.3	52.7	38.9-66.1
Dark	53.3	28.8-76.3	43.5	17.4-73.7	89.2	79.5-94.6	57.9	36.5-76.6
Total	63.0	51.2-73.4	46.5	34.3-59.1	48.9	36.4-61.6	55.5	44.6-66.0
General Total	55.7	45.7-65.2	42.6	32.2-53.6	46.8	37.8-56.1	50.1	40.2-59.9

*CI confidence interval

In 50.1% (95% CI: 40.2-59.9) of the population with HBP who reported being under treatment, BP was controlled. The prevalence of controlled HBP was higher in women compared to men, 55.5% (95% CI: 44.6-66.0) vs. 42.4% (95% CI: 31.5-54, 1), although the difference was not significant. Among men, the lowest prevalence of controlled HBP was found in the dark-skinned population aged ≥80 years, with 27.5% (95% CI: 8.4-61.0); followed by the clear-skinned population aged between 70 and 79 years, 32.7% (95% CI: 24.9 - 41.7). In women, the lowest prevalence of controlled HBP was observed in the medium-skinned population aged ≥ 80 years, 31.5% (95% CI: 22.4-42.3). In both men and women, the population aged between 70 and 79 years had the lowest prevalence of control, although the difference with other age groups was not significant (Table 3).

Only 1.4% of the population with HBP were not affiliated with any health regime, 2.4% were in a special regime; 45.0% in the contributory scheme; and 51.0% in the subsidized regime. 82.6% of the population with HBP had visited a physician at least once in the last month; and in this population, 55.2% (95% CI 43.1-66.8) had controlled HBP. In all skin groups, the population that reported visiting the physician in the last week had the highest prevalence of controlled HBP, without finding significant differences between the groups. On the contrary, only 32.5% (95% CI 26.8-38.7) of the population that did not visit the physician had controlled HBP; similar behavior was observed in all skin groups. The prevalence of controlled HBP was significantly higher in the population affiliated with the special health regimes, 85.8% (95% CI: 75.5 - 92.2); followed by the population affiliated with the contributory regime, 53.6% (CI 95% 41.6-65.1); and the subsidized one, 43.6% (95% CI 35.2-52.4). However, among the regimes that gathered the largest number of population, the subsidized and the contributory ones, no differences were found. Similarly, in each of the health insurance program no significant differences were found in the prevalence of control among populations, according to their skin group (Table 4). 

**Table 4:** Access to health care in the population aged 60 years or older with high blood pressure.

	Clear				Medium				Dark				Total			
	Non-controlled*		Controlled¥		Non-controlled		Controlled		Non-controlled		Controlled		Non-controlled		Controlled	
	%	CI	%	CI	%	CI	%	CI	%	CI	%	CI	%	IC	%	IC
Having no physician visits ‡	66	54.9-76.1	34	23.9-45.1	69	58.2-78.7	31	21.3-41.8	70	58.2-79.2	30	20.8-41.8	68	61.3-73.2	33	26.8-38.7
At least one physician visit ‡	45	33.7-56.7	55	43.3-66.3	45	31.6-59.3	55	40.7-68.4	42	19.6-69.1	58	30.9-80.4	45	33.2-56.9	55	43.1-66.8
**Percentage of affiliated persons in each health regimen**																
Contributive	48	37.3-58.9	52	41.1-62.7	43	28.9-58.9	57	41.1-71.1	44	17.7-74.2	56	25.8-82.3	46	34.9-58.4	54	41.6-65.1
Subsidized	55	47.0-63.0	45	37.0-53.0	60	48.5-70.1	40	29.9-51.5	53	35.7-69.4	47	30.6-64.3	56	47.6-64.8	44	35.2-52.4
Special regimes	12	5.6-24.3	88	75.7-94.4	33	6.8-76.9	67	23.1-93.2	6.3	0.6-44.5	94	55.5-99.4	14	7.8-24.5	86	75.5-92.2
Non-affiliation	67	39.5-86.2	33	13.8-60.5	73	46.1-89.4	27	10.6-53.9	44	18.9-72.0	56	28.0-81.1	68	48.2-82.3	32	17.7-51.8
Total	50	41.0-58.2	50	41.8-59.0	51	37.6-64.2	49	35.8-62.4	49	26.2-72.0	51	28.0-73.8	50	40.1-59.7	50	40.3-59.9

* Person with blood pressure ≥140/90 mm Hg

¥ Person with blood pressure <140/90 mm Hg

‡ Physician visits within the last 30 days

Overall, 21.8% of people with HBP reported moderate or intense physical activity at least three times a week. A higher percentage of men reported being active compared to women, 28.3% (95% CI 25.7-31.1) vs. 16.4% (95% CI 13.0-20.6). In contrast, the prevalence of current tobacco use was higher in men 15.4 (95% CI 12.2 - 19.2), compared with women 7.8% (95% CI 5.9 -10, 3). There are no differences in the consumption of fruits and vegetables between the sexes. Finally, a low prevalence of the adult population reported being a frequent consumer of alcohol 0.8% (95% CI 0.6 -1.0). (Table 5).

**Table 5:** Habits and cardiovascular risk factors in hypertensive population aged 60 years or older.

	Light		Medium		Dark		Total	
Physical Activity ƚ	%	CIŧ	%	CIŧ	%	CIŧ	%	CIŧ
Man	28.8	25.5-32.4	27.3	24.6-30.1	29.6	23.2-36.9	28.3	25.7-31.1
Woman	17.1	12.9-22.3	15.4	11.5-20.3	16	11.8-21.2	16.4	13.0-20.6
Total	21.9	18.8-25.4	21.3	19.4-23.4	23.4	19.5-27.7	21.8	19.5-24.3
								
**Smoking ¥**								
Man	13.3	10.3-17.2	15.2	11.4-19.9	23.5	17.6-30.5	15.4	12.2-19.2
Woman	6.9	4.9-9.8	7.2	5.2-9.8	15.5	11.0-21.5	7.8	5.9-10.3
Total	9.6	7.1-12.7	11.1	8.5-14.5	19.8	15.3-25.3	11.2	8.9-14.1
**Alcohol consumption ǂ**								
**Man**								
No consumption*	94.5	93.1-95.6	94.9	93.7-95.9	95.1	92.9-96.7	94.7	93.8-95.5
Regular consumption**	3.8	2.8-5.2	3.9	3.1-5.0	2.8	1.8-4.2	3.7	3.0-4.6
Frequent consumption ***	1.6	1.1-2.3	1.1	0.6-2.1	2.1	1.1-4.0	1.5	1.1-2.0
**Woman**								
No consumption	99	98.5-99.4	99.4	99.0-99.7	99.8	99.2-99.9	99.2	98.9-99.4
Regular consumption	0.8	0.5-1.2	0.3	0.1-0.9	0.1	0.0-0.2	0.6	0.4-0.9
Frequent consumption	0.2	0.1-0.3	0.2	0.1-0.4	0.2	0.0-0.9	0.2	0.1-0.3
**Total**								
No consumption	97.2	96.5-97.7	97.2	96.5-97.7	97.3	96.1-98.1	97.2	96.7-97.6
Regular consumption	2.0	1.6-2.5	2.1	1.7-2.7	1.5	1.0-2.3	2.0	1.6-2.5
Frequent consumption	0.7	0.5-1.1	0.6	0.4-1.1	1.2	0.7-2.2	0.8	0.6-1.0
**Fruits and vegetables intake**								
Man	63.1	58.5-67.5	63	55.8-69.7	70.2	66.6-73.5	63.9	59.6-68.1
Woman	74.5	70.5-78.1	70.9	64.7-76.4	73.9	68.4-78.8	73.3	69.4-77.0
Total	69.8	66.0-73.3	66.9	61.4-71.9	71.7	68.2-75.0	69.0	65.3-72.4

ŧ Confidence Interval

ƚ Perform moderate or intense physical activity, at least three times a week

¥ Active smoker

* Drink alcohol less than one day in a week, or do not consume alcohol

** Drink alcohol two or three times a week

*** Drink alcohol four or more days a week

¶ Fruits and/or vegetables intake at least twice a day

## Discussion

This is the first estimate of the prevalence of HBP in the adult Colombian population aged over 60 years, in which the ethnic condition is identified using the color palette tool. The prevalence of standardized HBP by age is 57.7%, being higher in women compared to men in all skin color groups. The difference is greater in groups older than 70 years, particularly in medium- and dark-skinned groups. The groups with the highest prevalence were white-skinned men and dark-skinned women, aged over 80 years.

In the Colombian population aged ≥60 years, the standardized prevalence of HBP by age was 57.7%: and increased with age. Women had a higher prevalence compared to men, 62.9% vs. 51.4% respectively. Similarly, in the SABE survey carried out in Bogotá (Colombia) in 2012, Cano-Gutiérrez *et al*. ^10^, found a prevalence of 56.9%; but unlike our results, the prevalence in women was only slightly higher 57.8%, compared to 55.4% men. It has been consistently observed that systolic blood pressure levels and the prevalence of HBP are higher in men compared to women until the age of 60, the age from which this pattern is reversed ^25^ ^,^ ^26^. In the United States, the age-adjusted HBP prevalence was 75% in women, and 65% in men aged 65 years or older between 2003 and 2006 ^25^. Similarly, prevalence studies conducted in England between 1994 and 2011 reported that from the age of 60, the prevalence of HBP in women is about ten percentage points higher in relation to that estimated for men ^26^. Finally, the prevalence reported in this study is similar to that estimated in other Latin American countries, such as Mexico, Puerto Rico, Peru and Venezuela, where there have been reported values ​​ranging between 50.0% and 70.0% for the population aged older than 60 years; being higher in women, compared to men ^3^ ^,^ ^27^.

We found no differences in the standardized prevalence of HBP among the groups classified as light-, medium- and dark-skinned groups, except for a slightly higher prevalence of HBP in dark-skinned women. Although in populations studies with the NHANHES survey, a higher prevalence of HBP in the black population was reported ^4^, the establishment of this condition as a risk factor for HBP has not been defined. Different studies have shown that black populations residing in rural areas in Africa had lower blood pressure levels than the migrant black offspring residing in the United States, and equally lower than the levels identified in the white population ^6^. Additionally, the black migrant population residing in England has shown a lower risk of developing cerebrovascular disease, compared to the native white population ^28^. Our findings are equally consistent with other surveys developed in Latin America. In a population survey carried out in Cienfuegos (Cuba), Orduñez *et al*. ^8^, found a similar prevalence of HBP in populations classified as white or black, 31.0%. The researchers performed the classification based on skin color. In a population residing in Puerto Rico, Gravlee et al. ^29^, found that the black population had higher levels of HBP, compared to the white population, but only in populations that belong to a high socio-economic stratum. In Brazil, Carvalho *et al*. ^7^, found that the black population was more likely to have HBP; however, the prevalence of HBP was similar in the two ethnic groups. These observations have led to the consideration of ethnicity as a social determinant for the development of HBP; and in general, for cardiovascular diseases ^30^ ^,^ ^31^. Therefore, the establishment of strategies to intervene the risk factors that contribute to the increase in HBP, such as salt intake and physical inactivity, as well as access to services, should be guaranteed for the entire population, emphasizing those residing in adverse environments.

Limited access to health services has been one of the factors that has contributed to poor control of HBP. In the current survey, it was found that the prevalence of control is lower in those who did not report visiting a physician at least once in the last month, in relation to those who did it. This observation was similar among the three skin color groups. These findings corroborate the importance of regular monitoring of people with chronic conditions in general; and particularly those with HBP ^32^. In Brazil, Macinko *et al*. ^33^, reported that the prevalence of controlled HBP increased from 33% to 57% when the population had received care through the primary care system. Brazil is also a multi-ethnic country, where interventions carried out at primary level have reduced the differences in cardiovascular morbidity between different ethnicities ^32^. These findings corroborate the importance of implementing public health strategies that equally reach all population groups. 

In the population with HBP, only 21.8% reported moderate to intense physical activity at least three times a week, the percentage being higher in men compared to women, 28.3% vs. 16.4%. Physical activity is one of the related factors, not only for the prevention of cardiovascular diseases ^34^ and falls, but also for n the overall well-being of the elderly ^35^. In this population group, physical activity has been associated with a lower risk of fractures, which can range between 12% and 29%. The older adult who performs physical activity additionally achieves greater independence and social integration. Our findings, therefore, show the need to strengthen public policies and strategies within health insurance plans to increase physical activity in the elderly.

### Limitations and strengths

This is the first estimate of the prevalence of HBP in Colombian population aged over 60 years, based on a population sample. However, our estimates have some limitations. Minority ethnic groups, particularly indigenous groups, may be under-represented since the sample size was based on the distribution by age and sex of the population aged over 60 years residing in the geographic regions defined for the analysis. However, the percentage of dark-skinned population (10.5%) was similar to the percentage of Afro-Colombian people identified in the last Colombian census ^12^. Memory bias could affect the estimate of HBP, which was mainly based on the information provided by the respondent. However, this method has been validated and is used in population surveys; additionally, the estimated prevalence is within the expected values ​​for this population group, both nationally and internationally ^5^ ^,^ ^10^ ^,^ ^11^ ^,^ ^22^. This bias could also compromise the estimation of other variables, such as eating habits and visiting health services. 

54.5%, 34.5% and 10.9% of the adult population was classified as light-, medium- and dark-skinned, identified with the use of the palette. This distribution is correlated with the data reported by the last Colombian census, which indicates that 10.6% of the population determines itself as Afro-Colombian or Afro-descendant ^12^. The color palette to classify skin color has been used for Colombia as a country included in an ethnographic survey in Latin America ^23^. In that survey, it was found that while 45% of the population recognized themselves as mestizos, this population was classified as light-skinned (40%) and medium-skinned (36%), using the color palette ^19^. We found a higher percentage of the dark-skinned population in low socio-economic strata, affiliated with the subsidized regime, and residing in rural areas compared to the population with light and medium skin color. Similarly, in a demographic study conducted by the United Nations in 2011, it was found that between 50% and 70% of the Afro-Colombian population was affiliated with the subsidized regime ^36^. The updated data on insurance affiliation in Colombia also shows that in territories with the highest proportions of Afro-Colombian population, health insurance is mostly provided through the subsidized regime ^37^. These data, therefore, show the consistency between the classification of the population obtained through the color palette and the ethnic distribution reported in Colombia.

## Conclusions

Our data show that the standardized prevalence of HBP, by age, is 57.7% for the Colombian population aged equal to or greater than 60 years. The prevalence was higher in women, 62.9%, compared to men 51.4%. No differences were found among ethnic groups, with the exception of a higher prevalence of HBP in dark-skinned women, compared to medium- and light-skinned women. 50.1% of the population with HBP had BP <140/90 mm Hg; higher control percentages were found for the population who regularly visited the doctor, and affiliated to the health system through special and contributory regimes. Greater efforts are needed to improve the control of HBP in adults aged over 60 years. In particular, it is required a greater strengthening of non-pharmacological interventions, such as physical activity, in order to improve not only the control of HBP, but in general, the well-being of the elderly.

## Supplementary Material

Ethnic-racial variable: Self-recognition questions and heteroclassification by color palette

**1. Ethnic self-recognition (DANE question)**

According to your CULTURE, PEOPLE or PHYSICAL TRAITS do you recognize yourself as …? *(Interviewer: read, select only one)*

- Indigenous
- Gypsy / ROM
- Raizal of the San Andrés and Providencia archipelago
- Palenquero of San Basilio
- Black, Afro-descendant or Afro-Colombian
- None of the above
- Does not respond
- Does not know

**2. Racial self-awareness (LAPOP question)**

You consider yourself a person ....

*(Interviewer: read, select only one)*

- Indigenous
- Black
- Mulatto
- White
- Mestizo
- Other, specify ____________________
- Does not respond
- Does not know

**3. Skin color. Heteroclassification**

*Interviewer: This item is done by observation, it is not a question, but consists of assigning a number of the color scale, from 1 to 11, to the skin color of the face of the person interviewed.*

*What is the skin color of the face of this person?*

Skin Color Palette Used in the 2010 Americas Barometer
