# Supplementary material for: Prevalence, awareness, treatment and control of high blood pressure in the elderly according to the ethnic group. Colombian survey
Source: Colomb Med (Cali). 2019 Jun 30;50(2):115–27. doi: 10.25100/cm.v50i2.4124 (PMC6774579; doi:10.25100/cm.v50i2.4124)
Supplement: Translation

# Translation: Prevalencia, conocimiento, tratamiento y control de la presión arterial elevada en el adulto mayor de acuerdo con el grupo étnico. Encuesta colombiana salud, bienestar y envejecimiento 2015

**Contribución del estudio**

**Table:** 

1) Por que se hizo este estudio?
Para estimar la prevalencia en Colombia, del aumento de la presión arterial en personas mayores de 60 años y relacinado con el color de la piel.
**2) Cuales fueron los resultados mas relevantes?**
Se hizo una encuesta nacional de las personas adultas en 2014. El 57.7% de los adultos mayores tuvieron un aumento de la presión arterial, con una mayor prevalencia en mujeres con el color de la piel negra (60.9%). El 93.9% de los adultos mayores con un aumento de la presión arterial estuvieron consientes de su diagnostico y entre estos el 50.1% estaban en tratamiento para el control de la presión. No hubo diferencias entre el color de piel y el control y el conocimiento de los problemas de presión entre los adultos mayores. Se determinó una baja actividad física entre todos los grupos de adultos
**3) Que significan los hallazgos?**
El aumento de la presion arterial present una alta prevalencia en los adultos colombianos; solo la mitad de ellos se controlaban la presión. Se necesita un mayor esfuerzo para la prevención del aumento de la presión arterial y para mejorar el manejo de esta afección en la población adulta. Se requiere, particularmente iniciativas para mejorar la actividad física entre esta población

## Introducción

La presión arterial elevada (PAE) es el principal de factor de riesgo cardiovascular a nivel mundial ^1^. Para el 2015, las estimaciones mundiales de la prevalencia de PAE (i.e.; presión arterial (PA) ≥140/90 mm Hg) en la población mayor a 18 años eran de 24.1% para hombres y 20.1% en mujeres ^2^. La PA se incrementa con la edad, particularmente en las sociedades occidentales, al punto que se ha reportado que cerca del 45% de la población mayor a 60 años sufre de PAE ^3^. La prevalencia difiere entre países, en algunos como México se ha reportado una prevalencia de 58.2%, mientras en la India la prevalencia estimada es de 32.3% en población mayor a 50 años. 

Diferencias en la prevalencia de PAE han sido reportadas entre grupos étnicos ^4^. La Encuesta de Salud de Estados Unidos- National Health and Nutrition Examination Survey (NHANES)- ha reportado en forma consistente una mayor prevalencia de PAE en población negra no hispánica en comparación con población blanca no hispánica 40.3% y 27.8% respectivamente en el año 2016) ^4^^,^^5^. Sin embargo, está mayor prevalencia no se observó en poblaciones nativas residentes en comunidades rurales en África. Por ejemplo, una menor prevalencia de PAE fue estimada en poblaciones nativas residentes en Ghana (4.6%) y Jamaica (6.8%) comparada con población afrodescendiente viviendo en Estados Unidos, (29.0%) ^6^. En forma similar, en Brasil se encontró una prevalencia ligeramente mayor de PAE en la población negra (25.8%) en comparación con la población blanca (24.1%) ^7^. Por el contrario, en Cuba se ha observado una prevalencia similar de PAE en poblaciones blanca y negra, 31%. ^8^. Todas estas observaciones sugieren inequidades en salud y aportan evidencia sobre la influencia de los determinantes sociales en el desarrollo de PAE, particularmente en la diáspora africana, en contraposición con las explicaciones genéticas asociadas a la presencia de PAE en esa población ^6^. 

En Colombia, un país considerado de medianos ingresos, la última prevalencia nacional de PAE estimada fue de 23.7% para la población entre 18 y 69 años en el año 2007 ^9^. La prevalencia se aumentó con la edad, siendo más alta en la población con edades entre 60 y 69 años, 58.9% ^9^. En forma similar, en Bogotá (SABE-Bogotá) se reportó una prevalencia de 56.9% en personas con edad ≥60 años ^10^. De otro lado, el estudio internacional de movilidad en el envejecimiento (IMIAS por sus siglas en inglés) encontró un prevalencia cercana del 70% de PAE en población con edades entre 64 y 75 años residentes en una ciudad intermedia localizada en la zona central del país ^11^. En ninguna de estas encuestas se consideraron las características étnicas en relación con la prevalencia de PAE. 

El Departamento Administrativo Nacional de Estadísticas de Colombia (DANE) ha establecido cuatro grupos étnicos por autoreconocimiento: indígena, afrocolombianos, raizal del archipiélago de San Andrés y gitano también denominado ROM. En particular, la población afrocolombiana definida como personas con reconocida ascendencia africana incluye a Palenqueros de San Basilio, negros y/o mulatos que representa el 10.6% de la población colombiana de acuerdo con el censo llevado a cabo en el 2007 ^12^. Las personas que no se identifican dentro de estos grupos se consideran sin autoreconocimiento étnico ^13^. La clasificación más frecuentemente utilizada para identificar etnia/raza se basa en el autoreconocimiento de las personas; sin embargo, se ha determinado que la clasificación externa por un entrevistador del color de piel tiene una estrecha correlación con las características étnicas y culturales y da cuenta de otros aspectos no considerados por el autoreconocimiento. Esa observación ha llevado al desarrollo del instrumento escala o paleta de colores para determinar la etnia/raza de una persona ^14^.

La población étnica afrodescendiente en Colombia reside principalmente en la región Pacífica y Caribe; el Departamento del Valle del Cauca cuenta con la mayor proporción de residentes identificados como afrocolombianos, 25.3% ^12^ mientras que la población indígena se encuentra principalmente en los departamentos de Orinoquía, Amazonia y Chocó. Los estudios de morbilidad y mortalidad relacionadas con PAE disponibles hasta el momento, no reportan resultados de acuerdo con las características étnicas de la población ^15^. En consideración con lo anterior, se analizaron los datos de la encuesta Colombiana de Salud, Bienestar y Envejecimiento (SABE) llevada a cabo en el año 2015 con el objeto de estimar la prevalencia de presión arterial elevada, el conocimiento, tratamiento y control de esta condición en la población con edad igual o mayor a 60 años en los grupos étnicos identificados en la encuesta. 

## Materiales y Métodos 

La encuesta SABE tuvo como objetivo establecer el estado de salud, bienestar y condiciones sociales de la población colombiana con edad mayor o igual a 60 años residente en comunidad. Los métodos de la encuesta fueron descritos previamente ^16^. En la encuesta se agruparon las variables en función de las categorías de factores determinantes del envejecimiento activo de la siguiente manera: económico, entorno social, entorno físico, factores personales, comportamiento y sistemas de servicios sociales y de salud. Además, dado que el envejecimiento activo incluye el papel de la cultura y el género como factores transversales que influyen en todos los demás determinantes, en el estudio SABE se incluyeron variables de análisis relacionadas con los indicadores demográficos, el estado socioeconómico, las regiones geográficas y raza/etnia. Para identificar el estrato socioeconómico, se utilizó la clasificación de estratos de los servicios públicos y el régimen de seguridad social se determinó de acuerdo con el tipo de afiliación reportada según las categorías régimen contributivo, subsidiado, de excepción o al régimen especial. Cuando manifestaba no estar afiliado, se indagó por las razones de no afiliación. 

Adicionalmente, se evaluaron hábitos como fumar cigarrillos (si fumó o no, si fumaba actualmente, tiempo en años en fumar, edad cuando empezó a fumar, cantidad de cigarros o tabacos que fuma o fumaba por día) y el consumo de alcohol (comportamiento actual y pasado); la actividad física actual se evaluó mediante una adaptación de la escala de actividades diarias de la vida diaria de Reuben ^17^. Los participantes se clasificaron en cuatro categorías según sus respuestas: deportistas frecuentes vigorosos, caminantes frecuentes, caminantes cortos frecuentes y personas que no hacían ejercicio con frecuencia (grupo sedentario). El estado nutricional se evaluó utilizando la versión original más larga de la Mini-Nutritional Assessment (MNA) ^18^. Las medidas de antropometría incluyen altura y peso corporal; se utilizaron un estadiómetro portátil (SECA 213) y una escala electrónica (escala de plataforma Kendall graduada).

De manera novedosa, además de las preguntas de autoreconocimiento étnico y racial incluidas tradicionalmente en este tipo de estudios, la encuesta utilizó una clasificación externa realizada por un entrevistador quien utilizó una paleta de colores (Ver material suplementario). El procedimiento para utilizar la paleta ha sido validado ampliamente en América Latina en las encuestas PERLA y LAPOP ^14^^,^^19^. Específicamente, el entrevistador clasificó en uno de los once colores incluidos en la paleta la cara de la persona entrevistada, siendo 1 el color más claro y 11 el color más oscuro. Para el análisis de este variable, la población fue categorizada en tres grupos a) color de piel claro (1 a 3), b) intermedio (4 a 5) y c) oscuro (6 a 11) de acuerdo con la escala de paleta.

La muestra se tomó con base en la población de referencia de la muestra maestra de las encuestas nacionales usando una técnica de muestreo multi-etápica, estratificada por conglomerados. En el análisis, se utilizaron diferentes pesos muestrales de acuerdo con la población estimada por el DANE para el año 2015 con el objeto de considerar las probabilidades de selección del diseño muestral. Se definió como persona con PAE a aquella con al menos uno de los siguientes criterios: a) referir que había sido diagnosticada con PAE por un trabajador de la salud, o b) referir que consumía medicamentos para reducir la presión arterial o c) tener una toma de presión arterial sistólica (PAS) mayor o igual a 140 y/o de presión arterial diastólica (PAD) mayor o igual a 90 mm Hg. La toma de la presión arterial se realizó en una submuestra de 5,760 personas mayores a 60 años y el valor utilizado para la clasificación fue el promedio de las últimas dos mediciones realizadas de acuerdo con el protocolo establecido ^16^^,^^19^^,^^20^. 

La prevalencia global de la PAE se estimó mediante el método de estandarización directo usando como referencia la población colombiana proyectada por el DANE para el año 2015 ^21^^,^^22^. La prevalencia de conocimiento acerca de sufrir PAE se estimó como la fracción entre aquellos que reportaron el diagnóstico por un trabajador de la salud y/o tomaban medicinas para reducir la PA y el total de la población clasificada con PAE ^23^. La prevalencia de personas en tratamiento se determinó entre la población clasificada con PAE mientras que la prevalencia de control se determinó como la relación entre las personas hipertensas en tratamiento con PA<140/90 mm Hg y el total de personas con PAE que reportaron tomar medicinas para reducir la PA ^20^. Las estimaciones se realizaron para cada uno de los grupos definidos con base en el color de piel. En los análisis se incluyeron solo categorías de variables con un error estándar (EE) relativo <30%. ^20^. La estimación de la varianza se realizó a través del método de linealización por series de Taylor ^24^. El análisis se realizó con el programa STATA v. 14.

## Resultados 

Un total de 23,694 adultos con edad ≥60 años fueron incluidos con base en la muestra estimada. De ese total, 54.5%, 34.5% y 10.9% fueron clasificados con color de piel claro, medio y oscuro respectivamente. De toda la población incluida, una mayor proporción se encontró en el grupo de edad entre 60 y 69 años, 56.9% (IC 95%: 55.5-58.3), y era de sexo femenino 54.5% (IC 95%: 53.3-55.8), residía en zona urbana 78.1% (IC 95%: 63.2-88.1), se ubicó en estratos socio-económicos bajos, específicamente en el estrato 1 el 20.8% (IC 95%: 19.5-39.3) y en el estrato 2, el 38.6% (IC 95%: 36.5-42.9) y residía en la zona central 27.0% (IC 95%: 9.3-57.2). Únicamente, el 2.2% (IC 95%: 1.6-3.0) de la población no estaba afiliada a ninguno de los planes del régimen colombiano de seguridad social. En comparación con la población clasificada con color de piel blanca o media, un mayor porcentaje de población clasificada con color de piel oscura se encontró en el estrato socio-económico bajo, vivía en área rural y estaba afiliada al régimen subsidiado. El 8% de la población con piel oscura reside en Cali, siendo la ciudad principal con mayor concentración de población con ese color de piel (Tabla 1). 

**Tabla 1:** Características de la población total incluida distribuida por el color de piel

Características		Claro		Medio		Oscuro		Total	
Sexo	n*	%	IC**	%	IC**	%	IC**	%	IC**
Hombre	10,112	41.0	39.1-42.8	49.8	46.1-53.4	54.2	50.2-58.2	45.5	44.2-46.7
Mujeres	13,582	59.0	57.2-60.9	50.2	46.6-53.9	45.8	41.8-49.8	54.5	53.3-55.8
**Edad (años)**									
60-69	12,101	56.0	54.1-57.9	58.5	56.9-60.1	56.3	52.4-60.1	56.9	55.5-58.3
70-79	7,720	30.5	28.7-32.4	29.4	27.8-31.1	31	26.9-35.4	30.2	29.1-31.4
80 y más	3,873	13.5	12.3-14.8	12.1	10.9-13.4	12.8	11.2-14.5	12.9	12.2-13.7
**Estado socio-económico**									
1	10,313	20.8	13.7-30.3	33	23.7-43.8	51.9	42.5-61.2	28.4	19.5-39.3
2	9,033	38.6	36.2-41.1	43	37.4-48.7	34.3	28.3-40.9	39.7	36.5-42.9
03-abr	4,181	37.7	28.8-47.4	22.8	17.4-29.3	13.6	8.7-20.5	29.9	22.0-39.3
05-jun	167	2.9	1.6-5.1	1.2	0.6-2.4	0.2	0.1-0.6	2	1.3-3.2
**Área**									
Urbano	17189	84.5	70.0-92.7	72.3	55.7-84.4	65	47.2-79.5	78.1	63.2-88.1
Rural	6505	15.5	7.3-30.0	27.7	15.6-44.3	35	20.5-52.8	21.9	11.9-36.8
**Región**									
Atlántico	6202	10.6	3.5-27.9	24.5	11.2-45.5	44.5	23.7-67.4	19.1	8.0-39.1
Oriental	3583	20.7	7.5-45.6	18.2	7.0-39.6	3.3	0.9-11.4	17.9	6.9-39.1
Orinoquia y Amazonia	1394	1.5	0.4-5.5	1.4	0.4-4.7	0.5	0.2-1.1	1.4	0.4-4.5
Bogotá	2003	23.4	3.6-71.2	11.2	1.6-49.9	3.7	0.5-23.4	17	2.5-62.0
Central	6351	29.5	9.8-61.8	27	9.5-56.6	15	4.2-41.4	27	9.3-57.2
Pacifica	4161	14.4	3.2-46.3	17.6	5.9-42.4	33.1	14.5-59.1	17.5	5.2-45.4
**Ciudades capitales**									
Barranquilla	544	3.1	0.4-20.5	4.1	0.6-24.8	5.5	0.8-30.3	3.7	0.5-23.2
Cali	923	7.4	1,0-38,3	5,7	0,8-30,8	8,1	1,3-37,0	6,9	1,0-35,5
Medellín	849	10.3	1,5-46,3	9,1	1,4-41,2	5,0	0,7-27,8	9,3	1,4-42,6
Otras	21378	79.2	51,0-93,3	81,1	58,1-93,0	81,4	60,4-92,6	80,1	55,2-92,9
**Régimen de afiliación en salud**									
Contributivo	8627	56.0	48,8-62,9	43,4	36,3-50,7	30,8	23,3-39,6	48,9	41,0-56,8
Subsidiado	14,160	39.5	32,2-47,3	52,9	45,7-59,9	64,9	56,8-72,3	46,9	38,9-55,1
Esquemas especiales	371	2.6	1,6-4,2	1,2	0,7-1,9	1,4	0,7-2,8	2	1,2-3,2
No asegurado	512	1.9	1,3-2,7	2,5	1,5-3,9	2,7	1,9-4,1	2,2	1,6-3,0

* N= número

Del total de personas incluidas, 13,360 personas fueron identificadas con PAE, 95.0% mediante entrevista y 5.0% a través de la toma de PA. Le prevalencia estandarizada por edad fue 57.7% (IC 95%: 55.2-60.2), 51.4% (IC 95%: 47.3-55.5) en hombres y 62.9% (IC 95%: 60.9-64.9) en mujeres. La prevalencia aumentó con la edad sin encontrarse diferencias significantes por color piel dentro de las categorías de edad estudiadas. Sin embargo, en el grupo de edad ≥80 años, los hombres con color de piel oscura presentan una prevalencia ligeramente menor en comparación con la población color de piel claro y por el contrario en las mujeres con color de piel oscura la prevalencia fue mayor. Entre la población con PAE y registro de presión de PA, los promedios de PAS y PAD fueron 141.0 mm Hg (134.1-147.9) y 80.0 mm Hg (78.0-82.0) respectivamente. Globalmente, la prevalencia fue significativamente mayor en mujeres en comparación con hombres, 62.9% (IC 95%: 60.9-64.9) vs 51.4% (IC 95%: 47.3-55.5) (Tabla 2).

**Tabla 2:** Prevalencia estandarizada* de presión arterial elevada por edad de presión arterial elevada para cada sexo y color de piel**.**

	60-69 años		70-79 años		≥80 años		Total	
Hombres	%	IC‡	%	IC	%	IC	%	IC
Claro	45.0	39.3-50.7	61.8	56.1-67.5	70.6	64.1-77.1	53.2	48.7-57.7
Medio	42.7	35.8-49.6	59.7	52.8-66.6	61.0	54.1-67.9	49.6	44.5-54.7
Oscuro	41.6	30.8-52.4	60.1	49.3-70.9	59.2	48.4-70.0	49.4	41.0-57.8
Total	43.7	38.4-49.0	60.8	55.9-65.7	65.6	58.9-72.3	51.4	47.3-55.5
**Mujeres**
Claro	58.3	55.8-60.8	65.5	63.0-68.0	71.9	66.8-77.0	62.5	60.5-64.5
Medio	55.2	48.9-61.5	67.4	61.1-73.7	76.9	71.6-82.2	61.7	57.8-65.6
Oscuro	65.0	57.2-72.8	73.6	65.8-81.4	80.4	74.3-86.5	69.9	63.6-76.2
Total	57.9	55.2-60.6	66.9	64.2-69.6	74.2	70.5-77.9	62.9	60.9-64.9
Total general	51.2	47.7-54.7	64.2	61.5-66.9	70.6	66.3-74.9	57.7	55.2-60.2
Niveles de presión arterial diastólica**								
	**Promedio**	**IC**	**Promedio**	**IC**	**Promedio**	**IC**	**Promedio**	**IC**
**Hombres**								
Claro	82.1	80.9-83.3	81.9	79.5-84.3	74.8	71.5-78.1	81.1	79.7-82.5
Medio	84.7	81.0-88.4	80.2	78.0-82.4	78.8	74.3-83.3	82.8	79.9-85.7
Oscuro	83.6	78.7-88.5	79.3	75.2-83.4	79.1	76.0-82.2	81.7	78.6-84.8
Promedio	83.3	81.5-85.1	81.1	79.1-83.1	76.8	73.9-79.7	81.8	80.2-83.4
**Mujeres**								
Claro	79.8	75.9-83.7	78.7	76.5-80.9	75.0	72.8-77.2	78.9	76.2-81.6
Medio	78.6	76.4-80.8	78.0	75.8-80.2	75.7	74.1-77.3	78.0	76.4-79.6
Oscuro	80.8	77.5-84.1	79.6	75.1-84.1	77.1	71.4-82.8	79.7	76.8-82.6
Promedio	79.5	76.2-82.8	78.5	76.7-80.3	75.4	74.0-76.8	78.7	76.3-81.1
Promedio general	81.2	78.7-83.7	79.7	77.9-81.5	76.0	74.0-78.0	80.0	78.0-82.0
Niveles de presión arterial sistólica **								
	**Promedio**	**IC**	**Promedio**	**IC**	**Promedio**	**IC**	**Promedio**	**IC**
**Hombres**								
Claro	136.9	128.9-144.9	149.6	141.4-157.8	145.9	132.2-159.6	142.3	133.5-151.1
Medio	144.2	136.6-151.8	141.4	134.1-148.7	140.9	129.3-152.5	143.1	136.8-149.4
Oscuro	141.9	131.7-152.1	145.8	135.2-156.4	152.9	141.3-164.5	144.5	135.3-153.7
Promedio	140.3	133.8-146.8	146.8	139.5-154.1	144.5	136.1-152.9	142.8	136.1-149.5
**Mujeres**								
Claro	135.7	127.7-143.7	143.9	135.7-152.1	142.5	132.3-152.7	139.0	131.4-146.6
Medio	132.7	125.1-140.3	147.5	141.2-153.8	150.4	145.7-155.1	139.5	132.4-146.6
Oscuro	152.7	135.3-170.1	142.0	131.0-153.0	141.5	132.7-150.3	147.3	135.7-158.9
Promedio	135.7	127.9-143.5	144.8	137.7-151.9	144.8	136.4-153.2	139.6	132.3-146.9
Promedio general	137.7	130.6-144.8	145.7	138.8-152.6	144.7	137.4-152.0	141.0	134.1-147.9

*Prevalencia estandarizada usando el método directo y la población colombiana 2015

** Niveles de presión en mm Hg para la submuestra

El porcentaje de personas hipertensas que conocían su condición fue de 93.9% (IC 95%: 90.8-95.9), sin encontrarse diferencia entre los sexos. En la población con color de piel claro y medio, los grupos con edad menor a 80 años tuvieron una menor prevalencia de conocimiento en comparación con la población mayor de 80 años. Por el contrario, una significante menor proporción de hombres reportó utilizar medicamentos antihipertensivos en comparación con las mujeres, globalmente, el 88.8% (IC 95%: 86.8-90.5) de población con PAE recibía tratamiento. Independiente del color de piel, el más bajo porcentaje de tratamiento se encontró en los hombres con edad entre 60 y 69 años. Globalmente, entre los hombres, el grupo con piel media presentó una menor prevalencia de tratamiento en relación con el grupo con color de piel clara. De otro lado, entre las mujeres, una menor proporción de aquellas con color de piel oscuro recibía tratamiento en comparación con mujeres de piel clara (Tabla 3). 

**Tabla 3:** Conocimiento, tratamiento y control de las personas con edad igual o mayor a 60 años y presión arterial elevada

Porcentaje de personas conocedoras de su condición hipertensiva								
	60-69		70-79		80 y más		Total	
	%	IC*	%	IC	%	IC	%	IC
**Hombres**								
Claro	90.4	82.0-95.1	91.4	85.3-95.1	91.0	85.6-94.5	90.8	85.5-94.3
Medio	87.1	78.3-92.6	94.3	90.4-96.6	96.0	91.5-98.2	90.8	85.2-94.4
Oscuro	92.8	84.9-96.7	94.5	88.0-97.5	93.4	81.7-97.8	93.5	88.9-96.3
Total	89.4	83.0-93.6	92.8	89.4-95.2	93.0	89.4-95.4	91.1	87.0-94.1
**Mujeres**								
Claro	94.4	87.6-97.6	95.5	93.0-97.2	97.5	95.2-98.7	95.3	91.9-97.3
Medio	97.9	96.5-98.8	94.0	86.4-97.5	94.3	86.9-97.6	96.1	93.1-97.8
Oscuro	95.2	86.9-98.4	98.2	93.6-99.5	100.0	0.0-0.0	97.0	92.4-98.8
Total	95.6	90.9-97.9	95.3	92.7-97.0	96.8	94.4-98.2	95.7	93.1-97.4
Total general	93.1	88.3-96.0	94.3	91.8-96.0	95.3	93.2-96.8	93.9	90.8-95.9
**Porcentaje de personas hipertensas en tratamiento antihipertensivo**								
	**%**	IC*	**%**	**IC**	**%**	**IC**	**%**	**IC**
**Hombres**								
Claro	80.8	76.2-84.7	91.6	87.2-94.6	93.7	88.2-96.7	86.7	84.1-88.9
Medio	79.8	74.4-84.3	81.8	67.5-90.6	80.8	63.4-91.1	80.6	74.4-85.7
Oscuro	79.6	68.4-87.6	92.4	86.1-96.0	77.1	53.6-90.8	84.2	77.6-89.1
Total	80.3	76.1-83.9	88.2	81.9-92.5	87.3	79.7-92.3	84.2	81.2-86.8
**Mujeres**								
Claro	90.3	85.3-93.7	95.5	93.5-96.8	96.6	95.2-97.6	93.0	90.6-94.9
Medio	89.0	86.5-91.1	92.2	84.4-96.3	92.2	87.7-95.2	90.6	88.2-92.5
Oscuro	82.0	74.0-87.9	93.2	87.8-96.3	96.5	93.1-98.2	88.2	83.4-91.8
Total	89.0	85.9-91.5	94.2	91.0-96.3	95.3	93.6-96.6	91.8	89.7-93.4
Total general	85.7	83.8-87.4	91.7	88.1-94.3	92.3	89.3-94.5	88.8	86.8-90.5
**Porcentaje de personas hipertensas en tratamiento y presión arterial<140/90 mm Hg**								
	**%**	IC*	**%**	**IC**	**%**	**IC**	**%**	**IC**
**Hombres**								
Claro	46.4	36.9-56.1	32.7	24.9-41.7	41.5	23.2-62.5	40.3	32.0-49.2
Medio	41.8	23.6-62.4	49.6	32.1-67.2	52.1	32.1-71.5	45.2	29.5-62.0
Oscuro	54.6	23.6-82.3	34.5	10.1-71.1	27.5	8.4-61.0	44.5	18.0-74.5
Total	45.4	33.1-58.2	37.6	27.7-48.6	43.4	30.2-57.5	42.4	31.5-54.1
**Mujeres**								
Claro	63.5	52.9-73.0	45.8	31.6-60.8	51.8	37.0-66.4	56.5	46.0-66.4
Medio	63.8	45.9-78.5	48.6	36.5-60.9	31.5	22.4-42.3	52.7	38.9-66.1
Oscuro	53.3	28.8-76.3	43.5	17.4-73.7	89.2	79.5-94.6	57.9	36.5-76.6
Total	63.0	51.2-73.4	46.5	34.3-59.1	48.9	36.4-61.6	55.5	44.6-66.0
Total general	55.7	45.7-65.2	42.6	32.2-53.6	46.8	37.8-56.1	50.1	40.2-59.9

*IC intervalo de confianza

En el 50.1% (IC 95%: 40.2-59.9) de la población con PAE que reportó estar en tratamiento se encontró la PA controlada. La prevalencia de PAE controlada fue más alta en las mujeres en comparación con los hombres, 55.5% (IC 95%: 44.6-66.0) vs 42.4% (IC 95%: 31.5-54.1) aunque la diferencia no fue significativa. Entre los hombres, las prevalencias más bajas de PAE controlada se encontraron en la población con edad ≥80 años y con color de piel oscura con 27.5% (IC 95%: 8.4-61.0) seguido de la población con edad entre 70 y 79 años y color de piel claro, 32.7% (IC 95%: 24.9-41.7). En las mujeres la más baja prevalencia de PAE controlada se observó en la población con color de piel media y con edad ≥ 80 años, 31.5% (IC 95%: 22.4-42.3). Tanto en hombres como en mujeres la población con edad entre 70 y 79 años tuvo la menor prevalencia de control aunque la diferencia con otros grupos de edad no fueron significantes (Tabla 3).

Únicamente el 1.4% de la población con PAE no se encontró afiliada a algún régimen de salud, el 2.4% se encontró en un régimen especial, el 45.0% en el régimen contributivo y el 51.0% en el régimen subsidiado. El 82.6% de la población con PAE había visitado al médico al menos una vez en el último mes y en esta población el 55.2% (IC 95%: 43.1-66.8) tenía la PAE controlad. En todos los grupos de piel, la población que reportó haber visitado al médico en la última semana tuvo las prevalencias más altas de PAE controlada sin encontrarse diferencias significantes entre los grupos. Por el contrario, solo el 32.5% (IC 95%: 26.8-38.7) de la población que no visitó al médico tuvo la PAE controlada; se observó un comportamiento similar en todos los grupos de piel. La prevalencia de PAE controlada fue significativamente mayor en la población afiliada a los esquemas especiales de salud, 85.8% (IC 95%: 75.5-92.2) seguido de la población afiliada al régimen contributivo 53.6% (IC: 95% 41.6-65.1), y subsidiado 43.6% (IC 95%: 35.2-52.4); sin embargo, entre los regímenes que reunieron el mayor número de población, el subsidiado y el contributivo, no se encontraron diferencias. Similarmente, en cada uno de los regímenes de aseguramiento, no se encontraron diferencias significantes en la prevalencia de control entre las poblaciones de acuerdo con su grupo de piel (Tabla 4). 

**Tabla 4:** Acceso al cuidado en salud en la población con edad igual o mayor a 60 años y presión arterial elevada.

	Claro				Medio				Oscuro				Total			
	No controlado*		Controlado¥		No controlado		Controlado		No controlado		Controlado		No controlado		Controlado
	%	IC	%	IC	%	IC	%	IC	%	IC	%	IC	%	IC	%	IC
No visita médica ‡	66.3	54.9-76.1	33.7	23.9-45.1	69.4	58.2-78.7	30.6	21.3-41.8	69.7	58.2-79.2	30.3	20.8-41.8	67.5	61.3-73.2	32.5	26.8-38.7
Al menos una visita médica‡	44.9	33.7-56.7	55.1	43.3-66.3	45.1	31.6-59.3	54.9	40.7-68.4	42.4	19.6-69.1	57.6	30.9-80.4	44.8	33.2-56.9	55.2	43.1-66.8
**Porcentaje de personas afiliadas en cada régimen de salud**																
Contributivo	48.0	37.3.58.9	52.0	41.1.62.7	43.3	28.9.58.9	56.7	41.1.71.1	44.0	17.7.74.2	56.0	25.8.82.3	46.4	34.9.58.4	53.6	41.6.65.1
Subsidiado	55.2	47.0.63.0	44.8	37.0.53.0	59.8	48.5.70.1	40.2	29.9.51.5	52.9	35.7.69.4	47.1	30.6.64.3	56.4	47.6.64.8	43.6	35.2.52.4
Esquemas especiales	12.2	5.6.24.3	87.8	75.7.94.4	32.9	6.8.76.9	67.1	23.1.93.2	6.3	0.6.44.5	93.7	55.5.99.4	14.2	7.8.24.5	85.8	75.5.92.2
No asegurado	66.9	39.5.86.2	33.1	13.8.60.5	72.9	46.1.89.4	27.1	10.6.53.9	43.6	18.9.72.0	56.4	28.0.81.1	67.6	48.2.82.3	32.4	17.7.51.8
Total	49.6	41.0.58.2	50.4	41.8.59	50.9	37.6.64.2	49.1	35.8.62.4	49.1	26.2.72	50.9	28.0.73.8	49.9	40.1.59.7	50.1	40.3.59.9

*Persona con presión arterial≥140/90 mm Hg

¥Persona con presión arterial<140/90 mm Hg

‡Visita médica en los últimos 30 días

Globalmente, el 21.8% de las personas con PAE reportaron realizar una moderada o intensa actividad física al menos tres veces por semana. Un mayor porcentaje de hombres reportó ser activo en comparación con las mujeres, 28.3% (IC 95%: 25.7-31.1) vs 16.4% (IC 95%: 13.0-20.6). Por el contrario, la prevalencia de consumo actual de tabaco fue mayor en hombres 15.4 (IC 95%: 12.2-19.2), en comparación con las mujeres 7.8% (IC 95%: 5.9-10.3). No se observan diferencias en el consumo de frutas y verduras entre los sexos. Finalmente, una baja prevalencia de la población adulta reportó ser consumidor frecuente de alcohol 0.8% (IC 95%: 0.6-1.0). (Tabla 5). 

**Tabla 5:** Hábitos y factores de riesgo cardiovascular en población hipertensa con edad igual o mayor a 60 años.

	Claro		Medio		Oscuro		Total	
Actividad física ƚ	%	ICŧ	%	ICŧ	%	ICŧ	%	ICŧ
Hombres	28.8	25.5-32.4	27.3	24.6-30.1	29.6	23.2-36.9	28.3	25.7-31.1
Mujeres	17.1	12.9-22.3	15.4	11.5-20.3	16.0	11.8-21.2	16.4	13.0-20.6
Total	21.9	18.8-25.4	21.3	19.4-23.4	23.4	19.5-27.7	21.8	19.5-24.3
**Smoking ¥**								
Hombres	13.3	10.3-17.2	15.2	11.4-19.9	23.5	17.6-30.5	15.4	12.2-19.2
Mujeres	6.9	4.9-9.8	7.2	5.2-9.8	15.5	11.0-21.5	7.8	5.9-10.3
Total	9.6	7.1-12.7	11.1	8.5-14.5	19.8	15.3-25.3	11.2	8.9-14.1
**Consumo de alcoholǂ**								
**Hombres**								
No consumo*	94.5	93.1-95.6	94.9	93.7-95.9	95.1	92.9-96.7	94.7	93.8-95.5
Regular consumo**	3.8	2.8-5.2	3.9	3.1-5.0	2.8	1.8-4.2	3.7	3.0-4.6
Consumo frecuente ***	1.6	1.1-2.3	1.1	0.6-2.1	2.1	1.1-4.0	1.5	1.1-2.0
**Mujeres**								
No consumo	99.0	98.5-99.4	99.4	99.0-99.7	99.8	99.2-99.9	99.2	98.9-99.4
Regular consumo	0.8	0.5-1.2	0.3	0.1-0.9	0.1	0.0-0.2	0.6	0.4-0.9
Consumo frecuente	0.2	0.1-0.3	0.2	0.1-0.4	0.2	0.0-0.9	0.2	0.1-0.3
**Total**								
No consumo	97.2	96.5-97.7	97.2	96.5-97.7	97.3	96.1-98.1	97.2	96.7-97.6
Regular consumo	2.0	1.6-2.5	2.1	1.7-2.7	1.5	1.0-2.3	2.0	1.6-2.5
Consumo frecuente	0.7	0.5-1.1	0.6	0.4-1.1	1.2	0.7-2.2	0.8	0.6-1.0
**Ingesta de frutas y verduras**								
Hombres	63.1	58.5-67.5	63.0	55.8-69.7	70.2	66.6-73.5	63.9	59.6-68.1
Mujeres	74.5	70.5-78.1	70.9	64.7-76.4	73.9	68.4-78.8	73.3	69.4-77.0
Total	69.8	66.0-73.3	66.9	61.4-71.9	71.7	68.2-75.0	69.0	65.3-72.4

ŧIntervalo de Confianza

ƚ Realizar actividad física moderada o intense al menos tres veces por semana

¥Fumador activo

*Tomar alcohol menos de un día al día en una semana o no consume alcohol

**Tomar alcohol dos o tres veces a la semana

***Tomar alcohol cuatro o más días a la semana

¶Ingerir de frutas y/o vegetales al menos dos veces al día

## Discusión 

Esta es la primera estimación de la prevalencia de PAE en población colombiana adulta mayor a 60 años en la cual se identifican la condición étnica usando la herramienta la paleta de colores. La prevalencia de PAE estandarizada por edad es 57.7%, siendo mayor en mujeres en comparación con hombres en todos los grupos de color de piel. La diferencia es mayor en los grupos con edad superior a 70 años particularmente en los grupos con piel color medio y oscuro. Los grupos con mayor prevalencia fueron los hombres con color de piel blanca y las mujeres con color de piel oscura con edades mayor a 80 años.

En la población colombiana con edad ≥60 años, la prevalencia estandarizada por edad de PAE fue de 57.7% y se incrementó con la edad. Las mujeres presentaron una mayor prevalencia en comparación con los hombres, 62.9% vs 51.4% respectivamente. Similarmente, en la encuesta SABE llevada a cabo en Bogotá (Colombia) en el año 2012, Cano-Gutiérrez et al. ^10^, encontraron una prevalencia de 56.9% pero a diferencia de nuestros resultados, la prevalencia en mujeres fue solo ligeramente mayor 57.8% en comparación con los hombres 55.4%. Consistentemente se ha observado que los niveles de presión arterial sistólica y la prevalencia de PAE son mayores en los hombres en relación con las mujeres hasta la edad de 60 años, edad a partir de la cual este patrón se invierte ^25^^,^^26^. En Estados Unidos la prevalencia de PAE ajustada por edad fue de 75% en mujeres y 65% en hombres con edades mayores o iguales a 65 años entre los años 2003 a 2006 ^25^. Similarmente, los estudios de prevalencia realizados en Inglaterra entre 1994 y 2011 reportaron que a partir de los 60 años la prevalencia de PAE en mujeres es cerca de diez puntos porcentuales mayor en relación con la estimada para los hombres ^26^. Finalmente, la prevalencia reportada en este estudio es similar a la estimada en otros países latinoamericanos como Méjico, Puerto Rico, Perú y Venezuela en donde se han reportado valores que oscilan entre el 50.0% y el 70.0% en población con edad mayor a 60 años, siendo mayor en mujeres en comparación con hombres ^3^^,^^27^. 

Nosotros no encontramos diferencias en la prevalencia estandarizada de PAE entre los grupos clasificados con color de piel claro, medio y oscuro excepto por una ligera mayor prevalencia de PAE en mujeres con color de piel oscuro. Aunque en estudios poblaciones con la encuesta NHANHES se reportó una mayor prevalencia de PAE en población negra ^4^, el establecimiento de esta condición como un factor riesgo para PAE no ha sido definido. Diferentes estudios han mostrado que poblaciones negras residentes en zonas rurales en África presentaron niveles de presión arterial menores a la descendencia negra migrante residente en Estados Unidos e igualmente menores a los identificados en población blanca ^6^. Adicionalmente, la población negra migrante residente en Inglaterra ha mostrado un menor riesgo de desarrollar enfermedad cerebro-vascular en comparación con la población blanca nativa ^28^. Nuestros hallazgos están igualmente acorde con otras encuestas desarrolladas en América Latina. En una encuesta poblacional llevada a cabo en Cienfuegos (Cuba), Orduñez *et al*. ^8^, encontraron una prevalencia similar de PAE en población clasificada como blanca y como negra, 31.0%. Los investigadores realizaron la clasificación con base en el color de piel. En población residente en Puerto Rico, Gravlee *et al*. ^29^, encontraron que la población negra tenía niveles más elevados de PAE en comparación con población blanca pero únicamente en poblaciones en estrato socio-económico alto. En Brasil, Carvalho *et al*. ^7^, encontraron que la población negra tenia mayor probabilidad de tener PAE, sin embargo la prevalencia de PAE fue similar en los dos grupos étnicos. Estas observaciones han llevado a la consideración de la etnia como un determinante social para el desarrollo de la PAE y en general de las enfermedades cardiovasculares ^30^^,^^31^. Por lo tanto el establecimiento de estrategias para intervenir los factores de riesgo que contribuyen al aumento de la PAE como la ingesta de sal y la inactividad física así como el acceso a los servicios se deben garantizar para toda la población haciendo énfasis en aquellas que residen en ambientes adversos. 

El limitado acceso a servicios de salud ha sido uno de los factores que ha contribuido a un pobre control de la PAE. En la actual encuesta se encontró que la prevalencia de control es menor en aquellas personas que no reportaron una visita médica en el último mes en relación con los que si lo reportaron. Esta observación fue similar en los tres grupos clasificados con base en el color de piel. Estos hallazgos corroboran la importancia del control regular de las personas con condiciones crónicas en general y particularmente con PAE ^32^. En Brasil, Macinko et al. ^33^, reportaron que la prevalencia de PAE controlada aumentaba de 33% a 57% cuando la población había recibido atención por el sistema de atención primaria. Brasil igualmente es un país multiétnico en donde las intervenciones realizadas a nivel primario han logrado reducir las diferencias en morbilidad cardiovascular entre las diferentes etnias ^32^. Estos hallazgos corroboran la importancia de implementar estrategias de salud pública que alcancen de manera equitativa a todos los grupos poblaciones.

En población con PAE únicamente el 21.8% reportó realizar actividad física moderada a intensa al menos tres veces por semana, siendo mayor el porcentaje en hombres en comparación con mujeres, 28.3 % vs 16.4%. La realización de actividad física en uno de los factores relacionados no solo en la prevención de enfermedades cardiovasculares ^34^, caídas sino el bienestar global del adulto mayor ^35^. En este grupo poblacional la realización de la actividad física se ha asociado con un riesgo menor de fracturas que puede oscilar entre el 12% y el 29%. El adulto mayor que realiza actividad física adicionalmente logra mayor independencia e integración social. Nuestros hallazgos muestran por lo tanto la necesidad de fortalecer las políticas públicas e igualmente las estrategias al interior de los planes de aseguramiento en salud tendiente a incrementar la actividad física en el adulto mayor. 

### Limitaciones y fortalezas

Esta es la primera estimación de la prevalencia de PAE en población colombiana mayor a 60 años basada en una muestra poblacional. Sin embargo, nuestras estimaciones tienen algunas limitaciones. Los grupos étnicos minoritarios, particularmente los indígenas, pueden estar sub-representados dado que el tamaño de muestra se basó en la distribución por edad y sexo de la población mayor a 60 años residente en las regiones geográficas definidas para el análisis. Sin embargo, el porcentaje de población con color de piel oscura (10.5%) fue similar al porcentaje de personas afrocolombianas identificadas en el último censo colombiano ^12^. El sesgo de memoria pudo afectar la estimación de la PAE la cual principalmente se basó en la información brindada por el encuestado. No obstante este método ha sido validado y es el utilizado en las encuestas poblacionales y adicionalmente la prevalencia estimada se encuentra dentro de los valores esperados para este grupo poblacional tanto a nivel nacional como internacional ^5^^,^^10^^,^^11^^,^^21^. Este sesgo también pudo comprometer la estimación de otros variables como los hábitos alimenticios y la visita a servicios de salud. 

El 54.5%, 34.5% y 10.9% de la población adulta fue clasificada como clara, media y oscura con base en el color de piel identificada con el uso de la paleta. Esta distribución se correlaciona con los datos reportados por el último censo colombiano en donde se indica que el 10.6% de la población se auto-determina como Afrocolombiana o afrodescendiente ^12^. La paleta de colores para clasificar el color de piel ha sido usada para Colombia como país incluido en una encuesta etnográfica en América-Latina ^21^. En esa encuesta se encontró que mientras el 45% de la población se auto-reconocían como mestizos, esta población era clasificada con color de piel claro (40%) y medio (36%) usando la paleta de colores ^19^. Nosotros encontramos que un mayor porcentaje de la población con color de piel oscuro en estratos socio-económicos bajos, afiliada al régimen subsidiado y residiendo en zonas rurales en comparación con la población con color de piel claro y medio. Similarmente, en un estudio demográfico realizado por las Naciones Unidas se encontró que entre el 50% y el 70% de la población afrocolombiana estaba afiliada al régimen subsidiado en el año 2011 ^36^. Los datos actualizados sobre aseguramiento en Colombia, igualmente muestran que en los territorios con las más altas proporciones de población afrocolombiana, el aseguramiento a salud es mayoritariamente brindado a través del régimen subsidiado ^37^. Estos datos por lo tanto muestran la consistencia entre la clasificación de la población obtenida a través de la paleta de colores con la distribución étnica reportada en Colombia. 

## Conclusiones

Nuestros datos muestran que la prevalencia estandarizada por edad de PAE es de 57.7% en población colombiana con edad igual o mayor a 60 años. La prevalencia fue mayor en mujeres, 62.9%, en comparación con hombres 51.4%. No se evidenciaron diferencias entre los grupos étnicos con excepción de una mayor prevalencia de PAE en mujeres con color de piel oscuro en comparación con las mujeres con color de piel medio y claro. El 50.1% de la población con PAE tenía la PA<140/90 mm Hg, mayores porcentajes de control se encontraron en la población con regular visita al médico y afiliada al sistema de salud a través de los regímenes especiales y contributivo. Mayores esfuerzos son necesarios para mejorar el control de la PAE en los adultos mayores a 60 años. Particularmente, se requiere un mayor fortalecimiento de las intervenciones no farmacológicas como la actividad física para mejorar no solo el control de la PAE sino en general el bienestar del adulto mayor. 

## Material suplementario

Variable étnico-racial: Preguntas de auto-reconocimiento y heteroclasificación por paleta de colores

**1. Auto-reconocimiento etnico (preguntas DANE)**

De acuerdo con su CULTURA, PUEBLO o RASGOS FÍSICOS usted se reconoce como…?

(Entrevistador: lea, seleccione solo una)

- Indigena
- Gitano / ROM
- Raizal del archipielago de San Andrés y Providencia
- Palenquero de San Basilio
- Negro, Afro-descendiente o Afro-Colombiano
- Ninguno de los anteriores
- No responde
- No sabe

**2. Identidad Racial (Preguntas LAPOP)**

Usted se considera una persona ....

*(Entrevistador: lea, seleccione solo una)*

- Indigena
- Negra
- Mulata
- Blana
- Mestiza
- Otro, especifique ____________________
- No responde
- No sabe

**3. Color de piel. Heteroclasificación**

***(Entrevistador: Este punto se diligencia por observación, no es una pregunta, consiste en asignar un número de la escala de colores, de 1 a 11, al color de la piel de la cara de la persona entrevistada)***

*¿Cuál es el color de piel de la cara de esta persona adulta mayor?*

Paleta Barómetro de las Américas utilizada en el 2010 para el color de la piel
